# Rheological Analysis of the Synthesis of High-Molecular-Weight Epoxy Resins from Modified Soybean Oil and Bisphenol A or BPA-Based Epoxy Resins

**DOI:** 10.3390/ma14226770

**Published:** 2021-11-10

**Authors:** Anna Sienkiewicz, Piotr Czub

**Affiliations:** Department of Chemistry and Technology of Polymers, Cracow University of Technology, Warszawska Str. 24, 31-155 Cracow, Poland; anna.sienkiewicz@pk.edu.pl

**Keywords:** modified soybean oil, vegetable oil modification, epoxy resin, epoxy fusion process, gelation, curing

## Abstract

The research undertaken in this work is one of the examples of the engineering of modern polymer materials. This manuscript presents studies on the gelation process which might occur during the synthesis of epoxy resin using the modified vegetable oil via the epoxy fusion process conducted in bulk. Based on obtained results we determined rheological parameters related to the properties of reacting mixture during the polyaddition process, especially before and after occurring the phenomenon of gelation (via (1) theoretical determination of the gel point using the degree of conversion of reactants before occurring the gelation process of reacting mixture and (2) experimentally—the dynamic mechanical properties such as storage modulus, G′; loss modulus, G″; and loss tangent, tg δ). Theoretical investigations show that for both systems: epoxidized soybean oil and bisphenol A (ESBO_BPA), as well as the hydroxylated soybean oil and low molecular weight epoxy resin (SMEG_EPR), theoretical values of the degree of conversion at the gel point are characterized by similar values (ESBO_BPA: *x_gel-theoretical_* = 0.620, *x_gel-theoretical_* = 0.620 and SMEG_EPR: *x_gel-theoretical_* = 0.614, *x_gel-experiment_* = 0.630, respectively), while the one determined based on the initial assumptions are greater than the above-mentioned (ESBO_BPA: *x_gel-assumed_* = 0.696 and SMEG_EPR: *x_gel-assumed_* = 0.667). Moreover, experimental studies in the viscoelastic fluid stage showed that the SMEG_EPR system is characterized by lower values of G′ and G″, which indicates lower elasticity and lower viscosity than the epoxidized derivative. It was found that alike during the conventional polyaddition reaction, both systems initially are homogeneous liquids of increasing viscosity. Wherein gradual increase in viscosity of the reaction mixture is related to the fusion of oligomer molecules and the formation of higher molecular weight products. In the critical stage of the process, known as the gelation point, the reaction mixture converts into the solid form, containing an insoluble cross-linked polymer.

## 1. Introduction

Epoxy resins as one of the commonly used thermosets are typically applied as adhesives in various applications, e.g., in the aerospace industry [1], surface coatings [2], construction composite materials [3], automotive and ship industries [4], packaging materials for electronic devices, as well as in medicine [5] or home fields. Among the most valuable properties of epoxies, resulting in huge demand in consumer markets, good adhesion to various substrates, high modulus, high-temperature performance, low shrinkage, and good corrosion resistance [6] are only a few to mention. However, on the other hand, these materials are characterized by high rigidity and brittleness, which are limiting their scope of applicability. There are numerous techniques used to overcome these disadvantages. One of them is the application of renewable resources for the synthesis and modification of epoxy resins. This trend is especially common recently when growing interest in bio-based polymer materials is seen more often due to their reduced environmental impact, unstable prices of petrochemical raw materials, as well as approaching prospect of their exhaustion. Among all available “green” raw materials, the application of vegetable oils is the most popular. Modified vegetable oils are usually used as reactive diluents [7] or to partially replace the traditional petroleum stocks in the synthesis and modification of polymer materials [8]. Our research team, based on the chemistry of the conventional epoxy resins synthesis from petrochemical raw materials, is conducting studies on novel epoxy materials obtained using modified vegetable oils that can partially replace petrochemical resources. Specifically, epoxidized oils can be substituted for low and average molecular weight epoxy resins, and hydroxylated ones can substitute for bisphenol A (BPA) in the fusion method of epoxy resins synthesis [8,9,10]. Additionally, the introduction of vegetable oils results in obtaining materials with better flexibility and a wider range of available cross-linking agents [11]. There are many groups of epoxy cross-linkers, for example, anhydrides [12,13], imidazoles [14,15,16], thiols [17,18], primary amines [19,20], and phenols [21]. However, with an application of vegetable oils for the process of the synthesis, this relatively large group of chemical compounds used as hardeners might be additionally extended by, e.g., isocyanates, which are usually applied in the polyurethanes technology [22,23]. Furthermore, it should be emphasized that the properties of the cured resins depend both on the type of epoxy compound and the type of curing agent employed. For the reason that curing agents greatly determine the properties of the resins, such as processability, electrical properties, and mechanical performance, many works focus on studying the curing agents and details of the crosslinking process itself [24,25,26]. 

The curing of thermosetting polymers is a complex process that affects the polymer’s rheological behavior due to changes in the physical properties of the material, such as the viscosity and the linear viscoelasticity complex modulus [27,28]. It must be stressed that changes in the rheological properties of a cured material are closely related to the phenomenon of gelation and vitrification, which are taking place during the crosslinking process. It is necessary to additionally point out here that the epoxy fusion process, used as a method of the synthesis of bio-based high-molecular-weight epoxy resins, involving reagents with diverse functionality (two for bisphenol A and an average of more than three for modified vegetable oil), exhibit a high possibility of obtaining a polymer with a branched structure or even cross-linking products. However, it was proven that skillful conduction of the epoxy fusion process in the presence of the lithium chloride (LiCl) catalyst, as well with comprehensive knowledge about all chemical and physical aspects of the process allows to obtain the product with designed characteristics, in a liquid form with high density, and without gelling of the reacting mixture.

Rheological studies have found various applications for the research on chemical and physical gelation processes of numerous polymeric materials [29,30,31]. The concept of gelation of material during the curing process is understood as a stage of the reacting system upon the certain degree of conversion, resulting in the incipient formation of an infinite network of cross-linked polymer molecules, resulting in an irreversible transformation of the sample from viscous liquid to a rubbery/elastic solid state [32]. According to Flory’s statistical theory, gel point exists when the first macromolecule with an infinitely large molecular weight, $\bar{M_{w}}\to \infty$, forms, and $\bar{M_{w}}$ is understood as weight-average molecular weight [33]. Hence, the material behaves already not as a liquid but not yet as a solid. At the gel point, the sample starts to show stiffness because of the build-up of the 3-D network [34]. While the system reaches the stage close of the gel point, the initially low shear viscosity progressively increases and an equilibrium modulus, G_e_, progressively increases, due to building the stronger gel network (η_0_ ∝ (p − pc)^−s^ for p < p_c_ and G_e_ ∝ (p − p_c_)^z^ for p > p_c_, where η_0_—shear viscosity, p—conversion factor, and p_c_—critical extent of reaction) [35]. However, the interval character of the rheological response of the system constitutes serious limitations for an accurate experimental determination of the sol–gel transition [36]. Gel point might be determined by the extrapolation of the steady-state viscosity to infinite viscosity [37]. However, this method is not accurate due to shear thinning with increasing viscosity and by the potential damage of the sample as gelation occurs. Gelation phenomenon, which might be studied using mechanical (Dynamic Mechanical Thermal Analysis, DMTA) [38], electrical (Dielectric Analysis, DEA) [39], and viscoelastic (rheometer) [40] properties, can be determined via the following rheological criteria: (1) the point where the elastic modulus, G′, and the viscous modulus, G″, are equal and tg δ = 1, where tg δ means mechanical loss factor, the relatively simple method of Tung and Dynes [41], unfortunately, does not consider the frequency dependence of the viscoelastic quantities; (2) the point at which tg δ becomes independent of the frequency; (3) the maximum in the tg δ; and (4) the crossing point between the tangent line of the elastic modulus curve and the baseline G′ = 0; and (5) the onset of the decrease in the rate of growth of the viscous modulus during the polymer cure [42]. There is a correlation between the gel point and the intersection point of the curves of storage and loss modulus. As indicated above, at the gel point G″ = G′ and tg δ = 1. In turn, in the liquid state, the viscous properties are predominant (G″ > G′ and tg δ > 1), whereas, in the solid state (G″ < G′ and tg δ < 1), the elastic properties are predominant.

Here, it is also worth mentioning other methods determining the gel point, such as the one proposed by Chambon and Winter [43], based on the theory that the relaxation modes are coupled. G(t) = St^n^, where S represents the gel strength factor and n, the relaxation exponent, are only necessary for the full characteristic of the linear viscoelastic properties of the incipient gel network (exceptions—short times, t < λ_0_ and high frequencies ω > 1/λ_0_). There is also known similar frequency dependence of the dynamic moduli, G′(ω) = k′ω^n^ and G″(ω) = k″ω^n^, for which the diagrams of the dynamic moduli at the gel point are parallel straight lines in a log–log plot and tg δ is independent of frequency. Dynamic frequency test is often applied to study the frequency dependence of G′ and G″ under small strain. Such scanning is used for the characterization of the network structure, where G′ mainly reflects the energy stored in the deformation process corresponding to elasticity, while G″ mainly reflects the energy lost corresponding to viscosity [44]. G′ and G″ and the intermediate frequency reflect the complexity of network structure for polymer without chemical cross-linking—the smaller the value is, the more complicated the network structure [45,46].

In this manuscript, we report studies on the rheological behavior of a reacting mixture in the epoxy fusion process carried out using the following: (*i*) biobased monomers (modified soybean oil), as well as (*ii*) monomers of functionality greater than 2: epoxidized soybean oil (functionality, f_ESBO_ = 3.6) and BPA or hydroxylated soybean oil (functionality, f_SMEG_ = 3.65) and low molecular weight epoxy resin (functionality equal 2) when approaching the gelling point. The research was concentrated on studying the rheological changes occurring while the curing process, before and during the gelation. In our opinion, the proper conducting the studies on such polyaddition processes, involving multifunctional reagents with a high probability of unexpected and sudden cross-linking, it is especially important to determine both the possibility of the formation of non-homogeneous polymer networks leading to occurring the phenomenon of gelation, as well as the gel time itself. We were also trying to apply the rheological investigations as a relatively simple tool for the estimation of the most optimal time for conducting such polyaddition process to obtain the final product of the highest possible average-molecular weight but still in the processable form.

## 2. Materials and Methods

The main substrate in the performed experiments was epoxidized soybean oil (ESBO, laboratory grade, Ergoplast EG, Boryszew, Poland, *EV* = 0.363 mol/100 g; with an average of 3.52 epoxide groups and 0.03 hydroxyl groups per 1 oil molecule).

### 2.1. Epoxidation of the Soybean Oil 

Epoxidation of the soybean oil was carried out in the system: hydrogen peroxide/acetic acid/sulphuric acid (POCh S.A., Gliwice, Poland reagent grade; 0.5 mol of acid, 1.5 mol of H_2_O_2_, and 0.03% of H_2_SO_4_ per 1 mol of unsaturated bonds in the oil). The reaction mixture was heated up to 55 °C, while stirring, H_2_O_2_ was added dropwise within 1 h. From the moment of adding the amount of H_2_O_2_, the reaction was carried out for a period of 6 h. After the reaction was completed, anhydrous calcium carbonate was added to the flask in a stoichiometric amount to H_2_SO_4_, to neutralize the used acid. Next, the reaction mixture was transferred to a separatory funnel, where the product (epoxidized soybean oil containing residues of the product of neutralization) was washed four times with a large excess of distilled water, and finally, the aqueous phase was separated from organic one, consisted of epoxidized soybean oil. The oil washed from the acid was vacuum distilled to remove residual solvent and water.

### 2.2. Hydroxylation of ESBO

In the first step oxirane rings in ESBO were open with the use of ethylene glycol (POCh S.A., Gliwice, Poland, pure). The reaction was carried out for 5 h at 110 °C and with a molar ratio of glycol to epoxy groups of 1.05. Additionally, 0.013 moles of sulfuric(VI) acid per 1 mole of epoxy groups in the epoxidized oil were used. Next, the reaction mixture was cooled and calcium carbonate was added to neutralize sulfuric acid. In the subsequent step, the organic phase was washed several times with hot distilled water and subjected to distillation under reduced pressure to remove residue of water and unreacted glycol from the mixture.

### 2.3. Epoxy Fusion Process

Modified vegetable oil (epoxidized or hydroxyl soybean oil, SMEG), bisphenol A (BPA, GE Cartagena, Murcia, Spain, 99.93%) or low molecular weight epoxy resin (EPR 0162, Hexion Specialty Chemicals, Inc., Columbus, OH, USA, *EV* = 0.584 mol/100 g), respectively, and LiCl (Merck, Warsaw, Poland, reagent grade; in the amount of 0.002 mol per 1 mol of OH groups) were involved in the fusion reactions. The process was carried out in the nitrogen atmosphere. The reaction mixture of modified soybean oil and BPA or EPR 162 was homogenized, followed by adding the LiCl and raising to the desired temperature of 160 °C. The duration of the process was established experimentally by monitoring the epoxy value of reacting mixture. The process was carried out until the assumed content of epoxy groups in the final product was reached. We obtained two polyaddition products: ESBO_BPA (via the reaction of epoxidized soybean oil and bisphenol A) and SMEG_EPR (throughout the fusion process of hydroxylated soybean oil and low molecular weight epoxy resin).

### 2.4. Iodine, Epoxy, and Hydroxyl Values of Vegetable Oil and Its Derivatives Were Evaluated According to Standards

- *Content of epoxy groups* (*epoxy value, EV*) was evaluated according to PN-87/C-89085/13 standard: samples were dissolved in HCl/1,4-dioxane (POCh, Gliwice, Poland, reagent grade) solution and titrated by NaOH/methanol (POCh, Gliwice, Poland, reagent grade) in a presence of cresol red as an indicator to the visual change of tint to purple;
- *The hydroxyl value* (*HV*), samples of soybean oil, its epoxidized and fusion derivatives were dissolved in the solution of catalyst [4(dimethylamino) pyridine in DMF, POCh, Gliwice, Poland, reagent grade] and acetic anhydride in DMF (POCh, Gliwice, Poland, reagent grade). Followed by intensive stirring for another 15 min and titration with KOH aqueous solution in a presence of thymolphthalein until the tint change from colorless to blue;
- *Iodine value* (*IV*), describing the content of unsaturated bonds, was determined with Hanus method according to PN-EN ISO 3961:2011 standard (samples of products were dissolved in chloroform (POCh, Gliwice, Poland, reagent grade), where iodine bromide in glacial acetic acid (POCh, Gliwice, Poland, reagent grade) was added and prepared solution was titrated with sodium thiosulphate in a presence of starch aqueous solution to the moment of its discoloration).

### 2.5. Gel Permeation Chromatography

The number-average molecular weight ($\bar{M_{n}}$), weight-average molecular weight ($\bar{M_{w}}$), and polydispersity (PI) of the obtained products were determined by Knauer Gel-permeation chromatography with two PL-Gel columns (300 × 7.5 mm^2^) with grain size 3 μm and MIXED-E pores, and a refractive index detector. The equipment was calibrated using standard polystyrene samples in the molecular weight range of 410 to 20,500 g/moL. The analyses were performed at 25 °C and the tetrahydrofuran dried over metallic sodium, distilled, and stabilized with BHT (2,6-bis(1,1-dimethylethyl)-4 methylphenol) was used as an as eluent (with eluent flow 0.8 mL/min).

### 2.6. Rheological Properties

Determination of the density of soybean oil derivatives and studies of the change of density during epoxy fusion process using epoxidized soybean oil and bisphenol A or hydroxylated soybean oil and EPR 0162 were made with Anton Paar MCR 302 rheometer, with a temperature control system type PPTD 200 and HPTD 200, in a plate-to-plate measuring system (upper plate with a diameter of 25 mm), at 25, 50, and 160 °C. The sample withdrawn from the prepared at 160 °C reaction mixture was transferred onto the rheometer gap between the parallel plates and subjected to sinusoidal shear stresses with a constant amplitude of 30 Pa and a frequency of 10 Hz.

## 3. Results and Discussion

### 3.1. The Synthesis of Bio-Based Epoxy Resin Using Soybean Oil Derivatives

**Figure 1:**
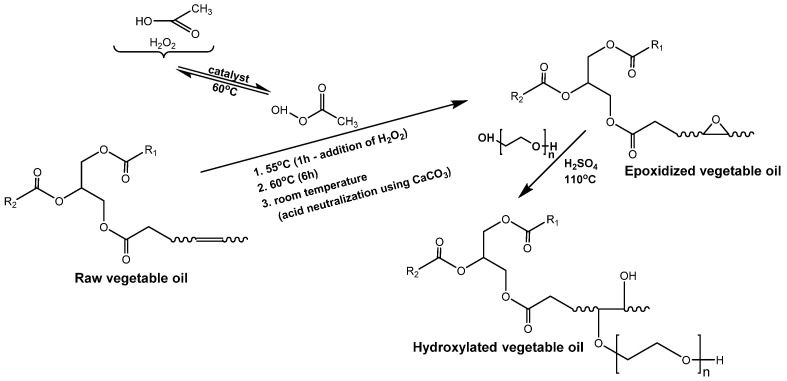
Synthesis of modified vegetable oil.

The synthesis of bio-based high molecular weight epoxy resins was carried out using modified soybean oil derivatives, such as epoxidized and hydroxylated soybean oil (Figure 1).

**Figure 2:**
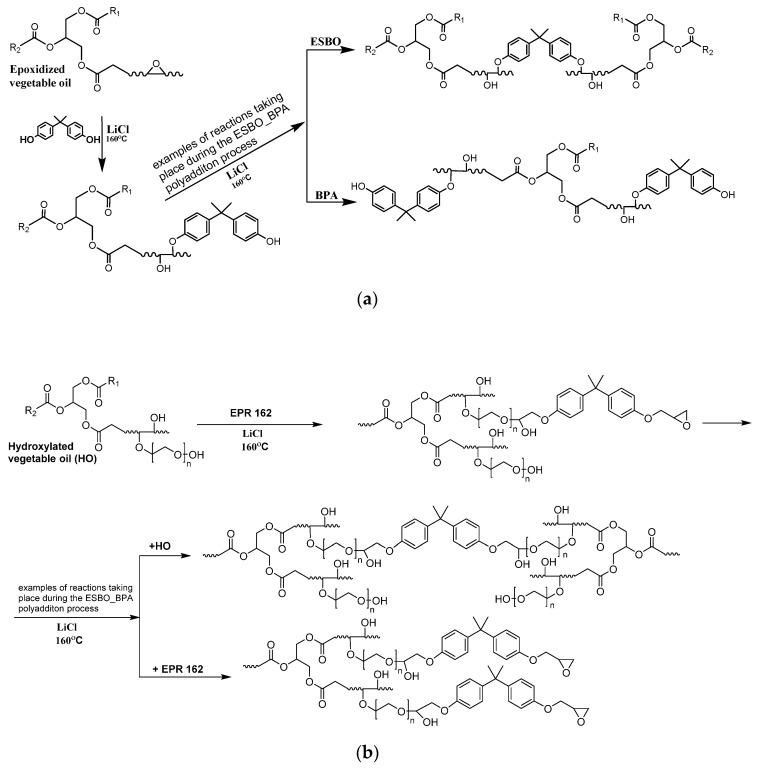
Fusion process of (**a**) epoxidized soybean oil and BPA and (**b**) hydroxyl soybean oil and EPR0162.

The epoxidation process of unsaturated bonds of soybean oil was conducted by Prilezaev method. The reaction was carried out in the system: acetic acid/hydrogen peroxide in the presence of sulfuric acid(VI) as a catalyst, using the organic peracid, formed in situ, as the actual oxidizing agent of this reaction. In turn, hydroxylated soybean oil was obtained in a two-step process: (1) epoxidation of unsaturated bonds in natural oil, and (2) the opening of newly formed oxirane rings by reaction with ethylene glycol. Next, soybean oil derivatives were used to obtain bio-based epoxy resins via the epoxy fusion process. Based on previously conducted studies [8] modified vegetable oils successfully can be applied to partially replace petrochemical raw materials used for the synthesis of epoxy resins. Therefore, epoxidized vegetable oils can replace low and average molecular weight epoxy resins, and hydroxylated ones can substitute for bisphenol A (BPA) in the conventional method of epoxy resins synthesis (Figure 2) [11,23].

The ESBO_BPA polyaddition product, characterized by *EV* = 0.118 mol/100 g, HV = 144 mg KOH/g, $\bar{M_{n}}$ = 2324 g/mol, and $\bar{M_{w}}$ = 6363 g/mol, was obtained in a process that took 18 h. In turn, the SMEG_EPR epoxy fusion process of hydroxyl soybean oil and a low-molecular-weight epoxy resin was carried out for 24 h resulting in a product with *EV* = 0.113 mol/100 g, HV = 160 mg KOH/g, $\bar{M_{n}}$ = 2267 g/mol, and $\bar{M_{w}}$ = 19,320 g/mol. Both syntheses were conducted in the presence of LiCl as a catalyst. The application of the catalyst was dictated by (1) the promotion of the reaction of phenol groups of bisphenol A/hydroxylated soybean oil with epoxy groups over the possible reaction of secondary hydroxyl groups (obtained by oxirane ring-opening) with epoxy groups, as well as (2) inhibition of branching reaction and in the same time prevention of sudden gelling of the reacting mixture. During the entire process samples of intermediate products were collected and subjected to the analysis of the content of functional groups by titration, along with mass spectroscopy and rheological analysis. A two-step change in the value of epoxide number and weight average molecular weight was found. Based on previously published results of performed studies [9,10], it could be expected that numerous reactions occur, including reactions: (i) between -OH groups of bisphenol A/hydroxylated soybean oil with oxirane rings of epoxidized soybean oil/EPR 0162 low-molecular-weight epoxy resin (depending on the reagents used: ESBO_BPA/SMEG_EPR epoxy fusion process, respectively), resulting in the formation of a bimolecular intermediate product containing one molecule of ESBO/EPR0162 and one molecule of BPA/SMEG (ESBO + BPA or SMEG + EPR, respectively), as well as (ii) of newly formed products with other molecules of ESBO/SMEG or BPA/EPR0162, causing the creation of a high molecular weight epoxy product.

### 3.2. The Rheological Studies of Epoxy Fusion Process Using Soybean Oil Derivatives

Within the tested range of shear stresses and shear velocities, both mixtures show a pseudoplastic character, which is distinguished by a decrease in the viscosity value with an increase in shear rate. In this case, shear strength contributes to untangling, stretching, and, parallel to the acting force, ordering of modified soybean oil macromolecule chains and low-molecular-weight epoxy resin.

**Figure 3:**
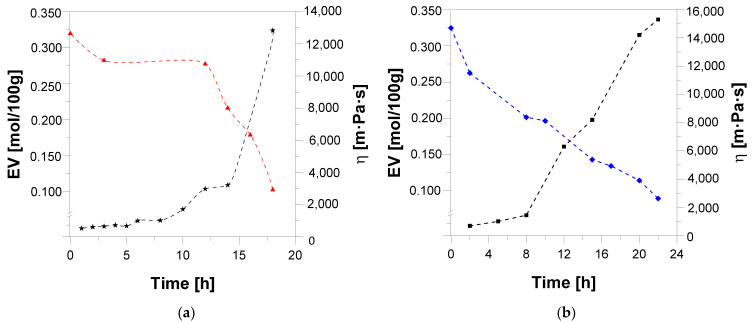
Change of EV and viscosity of the reaction mixture during the polyaddition process conducted in bulk via the fusion method: (**a**) ESBO_BPA and (**b**) SMEG_EPR.

ESBO_BPA and SMEG_EPR polyaddition products as viscoelastic liquids exhibit features of both solids (partial elastic recovery) and tacky liquids. Therefore, for samples taken during the polyaddition process of ESBO_BPA and SMEG_EPR, next to the determination of the content of epoxy groups, also the viscosity of the reaction mixture was studied. Based on obtained results (Figure 3) a typical, for the polyaddition reaction, course of viscosity changes was found [47,48].

**Figure 4:**
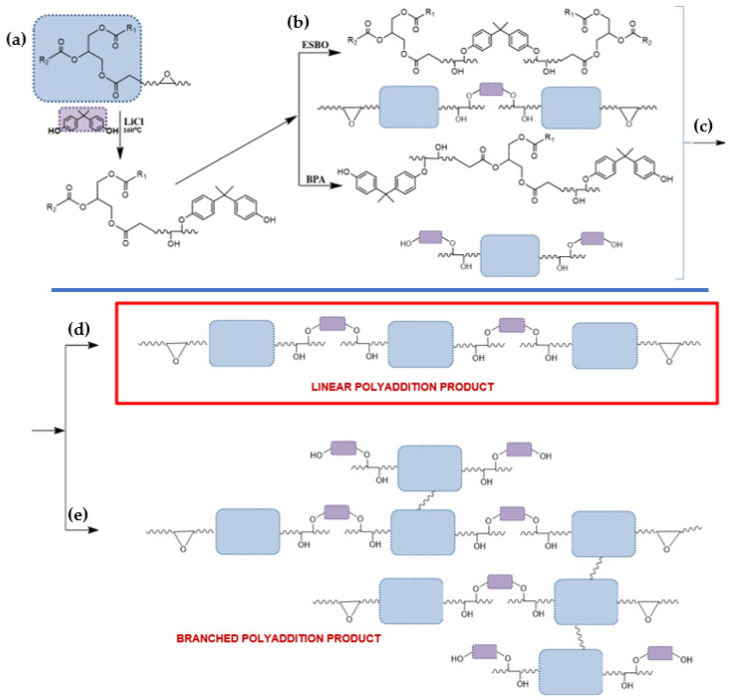
The ESBO_BPA epoxy fusion process: (**a**,**b**) 1st step—polyaddition reaction of epoxidized vegetable oil and BPA, (**c**) reaction of the dimer formed in the first stage of the polyaddition process, (**d**) formation of a linear fusion product, and (**e**) formation of a branched ESBO_BPA product.

**Figure 5:**
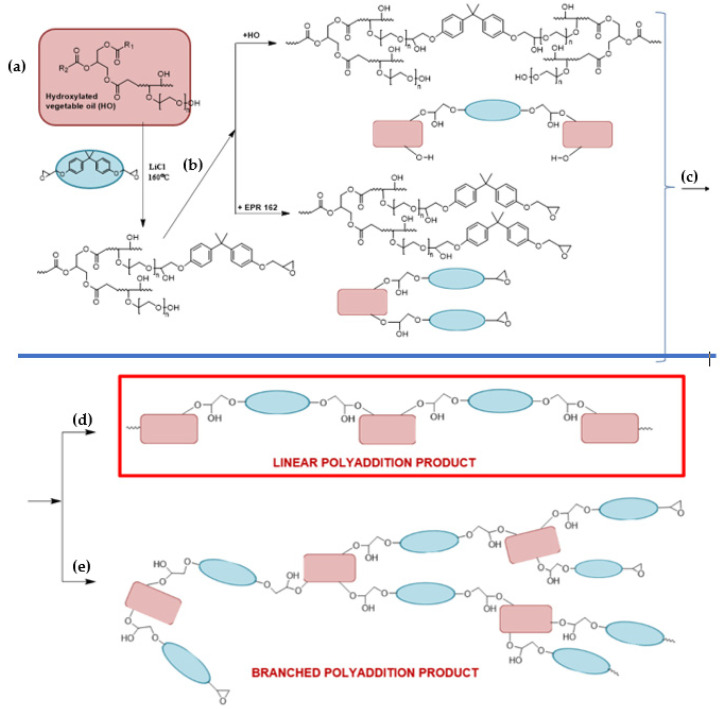
The SMEG_EPR epoxy fusion process: (**a**,**b**) 1st step—polyaddition reaction of hydroxyl soybean oil and low-molecular-weight epoxy resin, (**c**) reaction of the dimer formed in the first stage of the polyaddition process, (**d**) formation of a linear fusion product, and (**e**) formation of a branched SMEG_EPR_BPA product.

In this process, like in the case of a typical polymerization, initially, the reaction mixture is a homogeneous liquid; however, with the progress of the reaction, the increase of viscosity is observed [49,50]. This phenomenon is especially noticeable in the final stage of the reaction. In the initial stage of the polyaddition process, the mentioned gradual increase in the viscosity of the reacting mixture is related to the connection of oligomer molecules and the formation of products with a higher molecular weight (Figure 4 and Figure 5).

**Figure 6:**
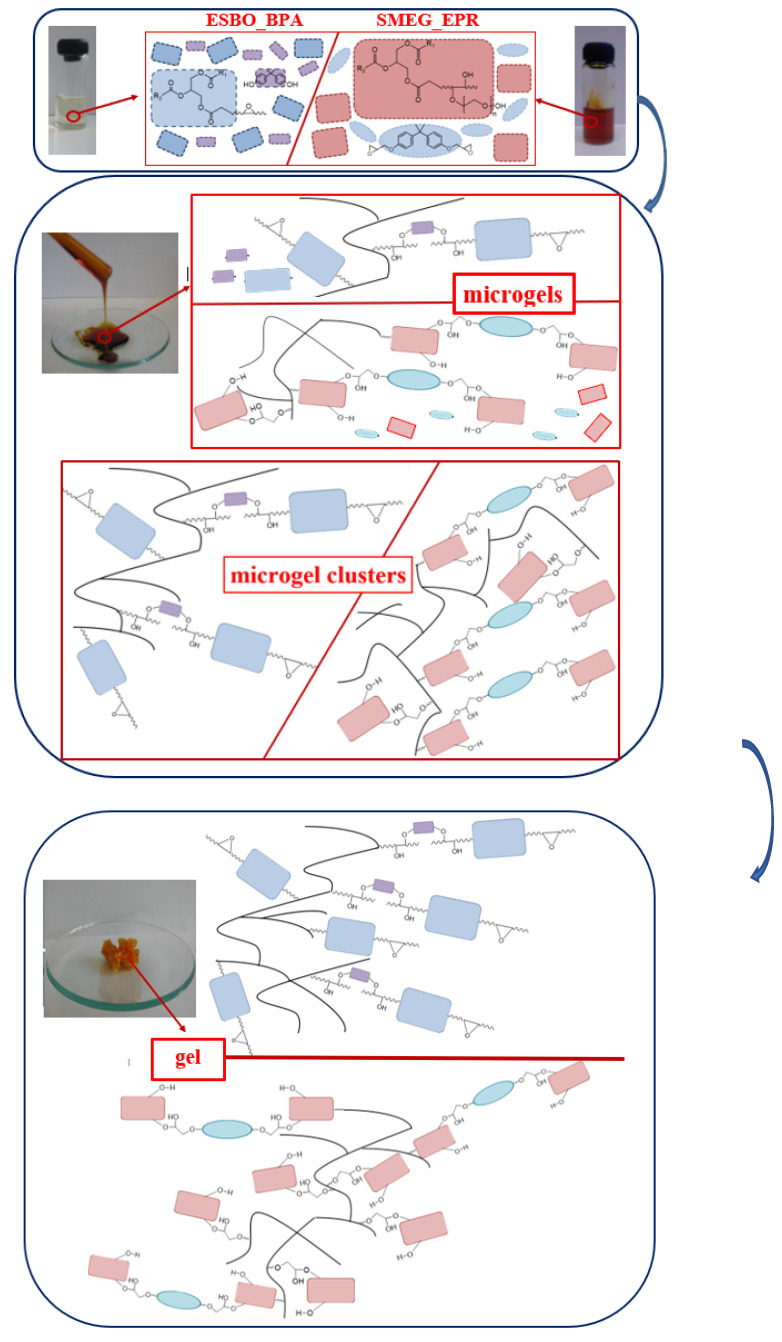
The course of changes, which are taking place during the polyaddition process from the beginning of the process until the gelation of the reaction mixture.

However, in the critical moment, which is called the gelation point, the reaction mixture converts into the solid form with insoluble cross-linked polymer (Figure 6).

**Figure 7:**
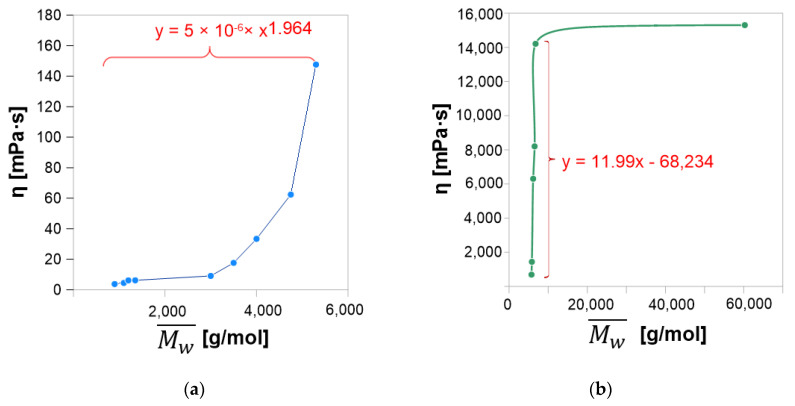
Change of viscosity and weight-average molar mass of the reaction mixture during the polyaddition process conducted in the bulk via the fusion method: (**a**) ESBO_BPA and (**b**) SMEG_EPR.

In a large assumption, gelation occurs when the molecular weight of the growing macromolecule is so significant that the reaction system might be described as the one made up of one large molecule. A gel network is composed of fractal clusters (flocs) of aggregated molecules or particles, which within time aggregate with each other. Its final structural architecture and macroscopic stiffness are the resultant of interfloc versus intrafloc links [51]. It is also not without significance that the rheological properties of polymers are closely determined by their molecular structure. Hence, polymeric materials are composed of molecules with different lengths, which distribution is determined by average-weight molecular mass, it was found that the comparison of zero-shear viscosity η_0_ with average-weight molar mass plays an important role in the structural analysis of polymeric materials. Hence, in the structural analysis of polymeric materials for linear materials the relationship η_0_ ∼ ${\bar{M_{w}}}^{a}$ applies, where exponent ‘a’ takes values of 3.4–3.8. In these polymeric systems, a slight change of ($\bar{M_{w}}\;$) has a great effect on η_0_, due to the significant power-law dependence of η_0_ on $\bar{M_{w}}\;$ [30]. Taking into consideration that dependence, Figure 7 presents the change of viscosity and average-weight molecular mass during the ESBO_BPA and SMEG_EPR process.

As it is shown on presented plots: the curve for the reaction ESBO_BPA does not indicate the formation only linear products—exponent “a” exhibits a lower value than those indicated in the literature (3.4–3.8). Within the first four hours of the process, the increase in the value of viscosity and molecular weight is relatively slow. It is probably related to the connection of the smallest oligomer molecules and the formation of larger groupings. Since the registered values of molecular weight are not higher than 1500 g/mol, this increase might be correlated with the connecting BPA molecules to the molecule of epoxidized soybean oil. As the reaction proceeds, the increase both in viscosity and the molecular weight is more noticeable. However, the equation η(${\bar{M_{w}}}^{a}$) for that part of the plot for ESBO_BPA process does not follow the power-law dependence, which is characteristic for the creation of only linear products. Simultaneously, as indicated in studies cited in the literature [30], it does not mean that the creation of such products in significant amounts does not proceed. Next to the formation of linear products, probably the process of branching and tangling of long chains takes place. The ESBO_BPA reacting system tends more towards branched structures. Such tendency is even more noticeable in the case of the reaction of hydroxylated soybean oil and low-molecular-weight epoxy resin, which where up to the 20th hour of conducting the process. The average molecular weight is not larger than 7000 g/mol, while the viscosity is sharply increasing from 685.07 to 14,208.0 mPa·s. It is worth pointing out here that both substrates of the SMEG_EPR process (hydroxylated soybean oil and low-molecular-weight epoxy resin) are characterized by long-chain structure, while in the ESBO_BPA process, modified vegetable oil, is a large molecule with long chains built up from fatty acids residues, but the BPA molecule is relatively small. Finally, in the last stage of the SMEG_EPR process, created oligomers connect with one another, resulting in an over an eightfold increase of average molecular weight within the next four hours of the process.

#### 3.2.1. Theoretical Determination of the Degree of Conversion at the Gel Point for the Polyaddition Process of Modified Soybean oil with BPA or EPR 0162, Carried out in Bulk, via the Fusion Process

Due to conducting the process in the system of reactants, characterized by a reactivity greater than 2, and therefore with an increased tendency of cross-linking, particular attention had been paid to the possibility of conducting polyaddition in a controlled manner and at the same time to apply the rheological investigations as a relatively simple tool for the estimation of the most optimal time for conducting such polyaddition process. That is why it is extremely important to estimate at what degree of conversion of reactants the gelation process of reacting mixture may occur. It is also important to study the course of the performed reaction when approaching a gelation point. Therefore, the gel point for the discussed polyaddition process using raw materials of natural origin was determined in two ways: (*i*) theoretically—calculated using Equation (1) and (*ii*) experimentally—based on rheological measurements. It is worth noting here that the conditions of the ideal course of polymerization are achieved when: the functional groups are characterized by similar reactivity, which does not change as the reaction proceeds.

ESBO_BPA. The theoretical value of the degree of conversion at the gel point (*x_gel-theoretical_*) for the synthesis of high-molecular-weight resins via the polyaddition process of epoxidized soybean oil with BPA is shown in Equation (1).

*x_gel_* = *r*[(*f_A_* − 1)(*f_B_* − 1)]^−1/2^, (1)

where: *x_gel_*—degree of conversion at the gel point, *f_A_*, *f_B_*—functionality of the substrate A and B, respectively, and *r* (equal *r* ≤ 1) is the ratio of the total number of functionalities of the substrate, which was used in excess to the total number of functional groups in the substrate used in excess, in the case of discussed experiment *r* = 1.

The theoretical value of the degree of conversion at the gel point (*x_gel-theoretical_*) for the synthesis of large-molecule resins by polyaddition reaction of epoxidized soybean oil with BPA is shown in Equation (2a,b).

*x_gel_* = *r*[(*f_ESBO_* − 1)(*f_BPA_* − 1)]^−1/2^, (2a)

*x_gel-theoretical_* = [(3.6 − 1) × (2.0 − 1)]^−1/2^ = 0.620, (2b)

where: *f_ESBO_*—functionality of epoxidized soybean oil, *f_ESBO_* = 3.6 and *f_BPA_*—functionality of bisphenol A, *f_BPA_* = 2.0.

Taking into account the above dependence, the theoretical EV was calculated for the reaction mixture at the gelation point (Equations (3) and (4)):

*EV_gel-theoretical_* = *EV*_0_ (1 − *x_gel-theoretical_*), (3)

*EV_gel-theoretical_* = 0.112 mol/100 g, (4)

where: *EV*_0_—starting epoxy number of the reaction mixture containing modified soybean oil and BPA, *EV*_0_ = 0.296 mol/100 g; *x_gel-theoretical_*—the theoretically determined value of the degree of conversion at the point of the gel, *x_gel-theoretical_* = 0.620; *EV_gel-theoretical_*—the theoretically determined value of the epoxy number at the gel point.

On the other hand, as mentioned earlier, the amounts of reagents for the discussed polyaddition reaction of epoxidized soybean oil with BPA was calculated assuming that the obtained final polyaddition product will be in a liquid form of high viscosity and the content of epoxy groups at the level of approximately 0.100 mol/100 g (*EV_final_product_* = 0.100 mol/100 g). Hence, the actual EV value, which will be characteristic for the reaction mixture at the time of gelation, should be lower than *EV_final_product_* (Equation (5)).

(5) $$\frac{m_{BPA}}{m_{EVO}}=\frac{M_{BPA}\cdot \left(EV_{EVO}-EV_{final\_product}\right)}{M_{BPA}\cdot EV_{final\_product}+200}$$

where: *m_BPA_*—the amount of bisphenol A, *m_EVO_*—the amount of epoxidized vegetable oil, *EV_EVO_*—the epoxy value of epoxidized vegetable oil, *EV_final_product_*—the estimated epoxy value of the product of epoxidized vegetable oil and bisphenol A and *M_BPA_*—a molar mass of BPA.

That is why, in relation with the above, for the calculation of the theoretical rate of conversion (Equation (6)) for the ESBO_BPA polyaddition process the approximate value of *EV_gel_* was assumed as 0.090 mol/100 g.

*x_gel-assumed_* = (*EV*_0_ − *EV_gel-assumed_*)/EV_0_ = 0.696, (6)

where: *EV*_0_—the initial epoxy value of the reaction mixture containing the modified soybean oil and BPA, determined experimentally *EV*_0_ = 0.296 mol/100 g; *x_gel-assumed_—*theoretically determined, based on initial and assumed EV, the value of the degree of conversion at the gel point for the ESBO_BPA polyaddition process; *EV_gel-assumed_*—the epoxy value of ESBO_BPA reaction mixture at the gel point, *EV_gel-assumed_* = 0.090 mol/100 g.

As a result of the polyaddition reaction, which was carried out in the lab environment, we obtained ESBO_BPA product with the content of epoxy groups at the level of *EV_final_* = 0.118 mol/100 g. Therefore, it was assumed that for this particular reaction the theoretical content of epoxy groups at the gel point was: *EV_gel-experiment_* = 0.111 mol/100 g and on this basis, the theoretical value of the degree of conversion at the gel point for the polyaddition process (*x_gel-experiment_*) is equal 0.625 (Equation (7)):

*x_gel-experiment_* = (*EV*_0_ − *EV_gel-experiment_*)/*EV*_0_ = 0.625, (7)

where: *EV*_0_—an initial epoxy value of the reaction mixture containing modified soybean oil and BPA; *EV_gel-experiment_*—epoxy value at the gel point, determined taking into account the content of oxirane groups in the final product.

Comparing the results of the relations presented above, it was found that the theoretical degree of gel point conversion, calculated for the performed ESBO_BPA polyaddition reaction (*x_gel-experimental_* = 0.625, according to Equation (7)) is similar to the theoretical conversion degree, which was calculated at the gel point and determined taking into account the functionality of the reactants participating in the reaction (*x_gel.-theoretical_* = 0.620—Equation (2)). In turn, the value of the conversion rate calculated at the point of the gel determined taking into account the initial assumptions is characterized by definitely higher value than the above-mentioned ones (*x_gel-assumed_* = 0.696). That is why, it can be anticipated that the theoretical model for determining the conversion rate at the gel point, taking into account the functionality of the reagents involved in the analyzed reaction, is closest to the realistically achievable value of this parameter. Additionally, considering both, the viscosity and the content of active functional groups of the final ESBO_BPA product, as well as the results of the above considerations, it can be assumed that the reaction in the analyzed experiment was interrupted at the best moment, which was very close to the point of gelation, and thus the final product contains as little as possible unreacted epoxy groups.

The SMEG_EPR polyaddition reaction carried out in bulk by the fusion method. Analogous, to the calculations presented above, theoretical analysis was also carried out for the synthesis of high-molecular-weight epoxy resins obtained by polyaddition of hydroxyl soybean oil and EPR 0162. Taking into account the functionality of the reagents involved in the discussed reaction, the theoretical value of the degree of conversion at the gel point was calculated as presented below (Equation (8)):

*x_gel-theoretical_ =* [(3.65 − 1) × (2.0 − 1)]^−1/2^ = 0.614, (8)

where: *f_SMEG_*—functionality of hydroxyl soybean oil, *f_SMEG_* = 3.65; *f_EPR_*—functionality of low-molecular-weight epoxy resin EPR 0162, *f_EPR_* = 2.0.

In the light of the above dependence, the theoretical value of EV was calculated for the reaction mixture at the gelation point (Equations (9) and (10)):

*EV_gel-theoretical_* = *EV*_0_ (1 − *x_gel-theoretical_*), (9)

*EV_gel-theoretical_* = 0.116 mol/100 g, (10)

where: *EV*_0_—the initial epoxy value of the reaction mixture containing the hydroxyl soybean oil and EPR 0162, *EV*_0_ = 0.300 mol/100 g; *x_gel.-theoretical_*—theoretically determined value of the degree of conversion at the point of the gel, *x_gel-theoretical_ = 0.606*; *EV_gel-theoretical_*—the theoretically determined value of the epoxy number at the gel point.

As before, it was assumed that the content of epoxy groups in the reaction mixture at the time of gelation should be less than the assumed value of the final *EV* (*EV_ER_* = 0.100 mol/100 g, Equation (11)).

(11) $$\frac{m_{HO}}{m_{ER}}=\frac{561\cdot \left(EV_{ER}-EV_{final\_product}\right)}{561\cdot EV_{final\_product}+HV_{HO}},$$

where: *m_HO_*—the amount of hydroxyl vegetable oil, *m_ER_*—the amount of low or average-molecular weight epoxy resin, *EV_ER_*—an initial epoxy value of low or average-molecular weight epoxy resin, *EV_final_product_*—estimated epoxy value of the product of hydroxyl vegetable oil and low or average-molecular weight epoxy resin, *HO*—hydroxyl value of modified vegetable oil.

Accordingly, to calculations of the theoretical degree of conversion (Equation (12)), for the SMEG_EPR process the value of *EV_gel-assumed_* was anticipated (*EV_gel-assumed_* = 0.090 mol/100 g):

*x_gel-assumed_* = (*EV*_0_ − *EV_gel-assumed_*)/*EV*_0_ = 0.667, (12)

where: *EV*_0_—an initial epoxy value of the reaction mixture containing hydroxyl soybean oil and EPR 0162, experimentally determined *EV*_0_ = 0.300 mol/100 g; *x_gel-theoretical_*—the theoretically determined value of the degree of conversion at the gel point for the polyaddition process SMEG_EPR, of which *EV*_0_ = 0.300 mol/100 g, *EV_gel-assumed_*—an epoxy value of the SMEG_EPR reaction mixture at the gel point, *EV_gel-assumed_* = 0.090 mol/100 g.

In the polyaddition process of hydroxyl soybean oil and EPR 0162 the product SMEG_EPR was obtained with the content of epoxy groups at the level of *EV_final_product_* = 0.113 mol/100 g. Therefore, for the discussed reaction the theoretical content of epoxy groups in the final polyaddition product was assumed at the *EV_gel-experiment_* = 0.111 mol/100 g. On this basis, the theoretical value of the degree of conversion at the gel point was determined as (Equation (13)):

x*_gel-experiment_* = (*EV*_0_ − *EV_gel-experiment_*)/*EV*_0_ = 0.630, (13)

where: *EV*_0_—the initial epoxy number of the reaction mixture containing the hydroxylated soybean oil and EPR 0162; *x_gel-theoretical_*—the theoretically determined value of the degree of conversion at the gel point for the SMEG_EPR polyaddition process for which *EV*_0*-experiment*_ = 0.300 mol/100 g; *EV_gel-experimental_*—epoxy number at the gel point, determined taking into account the content of oxirane groups in the final product.

Based on obtained results, it can be noted that alike for the reaction ESBO_BPA, discussed above, for studied SMEG_EPR polyaddition reaction theoretical values of the degree of conversion at the gel point *x_gel-theoretical_* and *x_gel-experiment_*, which were calculated using Equations (8) and (13), are characterized by similar values (*x_gel-theoretical_* = 0.614 and *x_gel-experiment_*= 0.630, respectively), while the one determined based on the initial assumptions, is definitely greater than the above-mentioned (*x_gel-assumed_* = 0.667). At the same time, it is worth noting that for the product SMEG_EPR, despite the similar to ESBO_BPA content of epoxy groups (respectively *EV*_SMEG_EPR_ = 0.113 mol/100 g and *EV*_ESBO_BPA_ = 0.118 mol/100 g) and theoretically similar potential values of the conversion rate, we register higher value of viscosity (respectively: η_ESBO_BPA_ = 12,836 Pa·s and η_SMEG_EPR_= 15,300 Pa·s). Among others, mentioned parameter, plays an important role at the stage of the preparation of the composition, especially in the moment of mixing with a chosen hardener. Taking into account the application point of view, it is important that the final polyaddition product is in the form of a liquid with a viscosity allowing for good homogenization with the hardener.

#### 3.2.2. Analysis of the Changes of the Rheological Parameters That Occurred during the Polyaddition Process of Modified Soybean Oil with BPA or EPR0162

In the next stage, for the analyzed polyaddition processes, an attempt to determine the dynamic mechanical properties (storage modulus, loss modulus, and the loss tangent) was made. For this purpose, the sample withdrawn from the prepared at 160 °C reaction mixture was transferred onto the rheometer gap between the parallel plates and subjected to sinusoidal shear stresses with a constant amplitude of 30 Pa and frequency of 10 Hz. We recorded changes in G′, G″, tg δ, and the real value of complex dynamic viscosity.

1. ESBO_BPA Polyaddition Reaction, Carried out in Bulk, using the Epoxy Fusion Method

**Figure 8:**
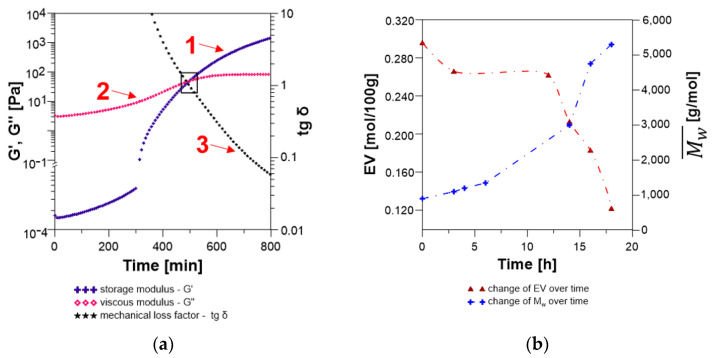
(**a**) Changes in the storage modulus (curve 1), loss modulus (curve 2), and the loss tangent tg δ (curve 3) over time for the polyaddition process of epoxidized soybean oil with bisphenol A; (**b**) changes in oxirane group content (EV) and molecular weight during the process ESBO_BPA.

Initially, the ESBO_BPA reaction system is in the form of a viscoelastic liquid (Figure 8).

However, as the reaction progresses, with the increasing molecular weight of the resulting polyaddition product, a gradual increase in the value of the loss modulus is observed (curve 2; the increase from 3.33 to 82.9 Pa). Thus, the value of the energy converted into heat, which is irretrievably lost from the system, also increases. At the same time, very low values of storage modulus are observed (curve 1), which for low frequencies of shear stresses (from 1.7 × 10^−4^ Pa) are close to 0. The point of intersection of G′ and G″ curves at t = 490 min determine the value of the loss factor tg δ = 1, thus the gel point of the reaction mixture. In turn, after crossing through the gel point for the analyzed reacting system, we observed G′ > G″ and tg δ < 1. In consequence, in the final stage, where the reaction mixture acquires the characteristics of a condensed solid, there is a significant increase of G′ values, until reaching high values, which are typical for the hardened product. The gelation time of the reaction mixture determined based on the above analysis was 8.2 h. It is important to note here, that for the reaction carried out in the flask, obtained value, is only the time when the content of oxirane groups is at the level of 0.260 mol/100 g, so definitely far away from the designated theoretical value of *EV* at the gel point. As it was mentioned before, polyaddition reaction of epoxidized soybean oil with bisphenol A was carried out for 18 h and during that time no gelling phenomenon was observed and the final product was characterized by *EV* = 0.118 mol/100 g. On the other hand, rheological analysis towards the determination of the gelation time of the reaction medium seems very important in light of different polyaddition products and allows to theoretically determine which of the analyzed systems shows a greater tendency for faster cross-linking.

2. The SMEG_EPR Polyaddition Reaction Carried out in Bulk by the Fusion Method

**Figure 9:**
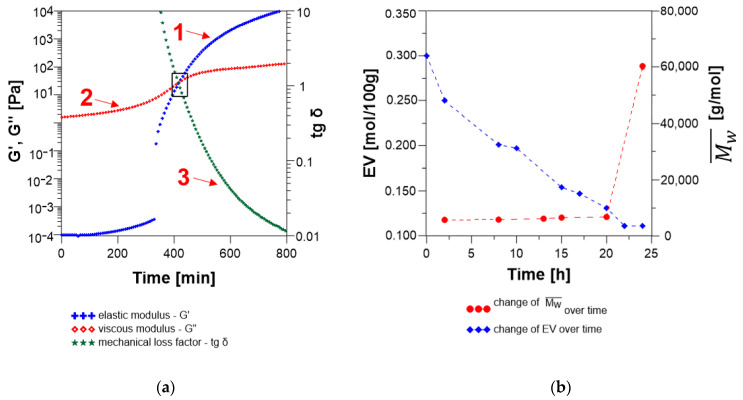
(**a**) Changes in the storage modulus (curve 1), loss modulus (curve 2) and the loss tangent tgδ (curve 3) over time for the polyaddition process of hydroxyl soybean oil with low-molecular-weight epoxy resin EPR0162; (**b**) changes in oxirane group content (EV) and molecular weight during the process SMEG_EPR.

The occurrence of three characteristic stages of changes in rheological parameters (Figure 9) was also found in the case of the polyaddition reaction of hydroxylated soybean oil with EPR 0162 carried out in bulk by the fusion method.

As the molecular weight of the resulting SMEG_EPR product increases, an increase in the loss modulus and very low values of the storage modulus (close to 0 for low frequencies of shear stresses) are observed. Moreover, as the reaction progressed, the increase in the values of G′ and G″ was observed, until the mixture becames gelatinized at time t = 410 min.

Additionally, the change of the storage modulus G′ might be used to determine the rheological degree of conversion β (Equation (14)) [33] at the gel point:

β = (G′_t_ − G′_0_)/(G′_∞_ − G′_0_), (14)

β_ESBO_BPA_ = (42.305 − 1.7 × 10^−4^)/(1760.2 − 1.7 × 10^−4^) = 0.0240, (15)

β_SMEG_EPR_ = (19.658 − 9.8 × 10^−5^)/(11,330.0 − 9.8 × 10^−5^) = 0.0017, (16)

where: G′_∞_ is the storage modulus of the completely cured resin, G′_0_ is the storage modulus at the beginning of the reaction, and G′_t_ is the storage modulus at time t. From the rheological point of interpretation reacting system ESBO_BPA exhibit a larger degree of conversion than SMEG_EPR. 

**Figure 10:**
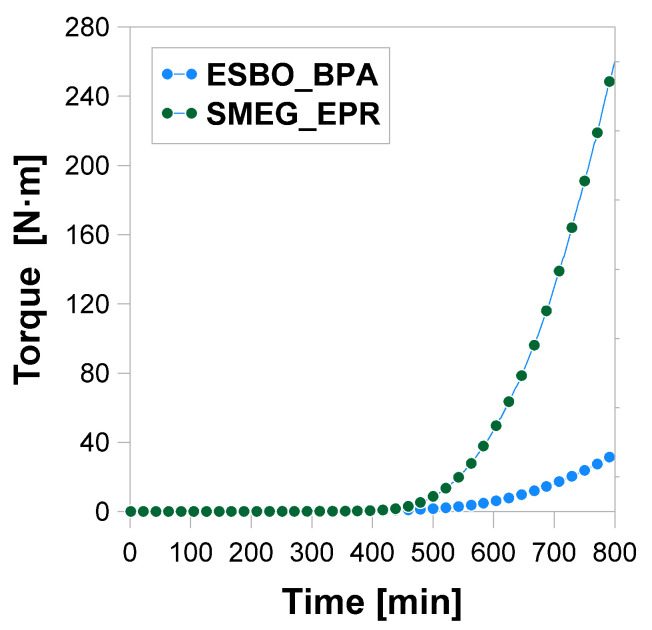
Torque as a function of processing time.

For both polyaddition products, an analysis of torque as a function of processing time was also performed (Figure 10).

Up to approximately 400 min, the increase in the value of torque was rather slow. Recorded values changed from 0.076 up to 0.500 N·m and 0.036 to 0.586 N·m for ESBO_BPA and SMEG_EPR, respectively. However, for both processes, starting approximately around 400 min, a sharp increase begins, which means that the chain extension/branching reactions occur in significant amounts during both of the studied reactions [52,53]. Simultaneously, the rate of the mentioned increase of torque values for the process of the SMEG_EPR is much more intense and achieves much higher values than those for ESBO_BPA. Bearing in mind the extensive structure of initial substrates, hydroxylated soybean oil, and low-molecular epoxy resin, it might be assumed that during the SMEG_EPR process the reacting system mainly forms branched products. Probably, such an architecture of emerging products with an increasing average molecular weight, results in much faster, compared to the ESBO_BPA, time of gelation for the SMEG_EPR process.

**Figure 11:**
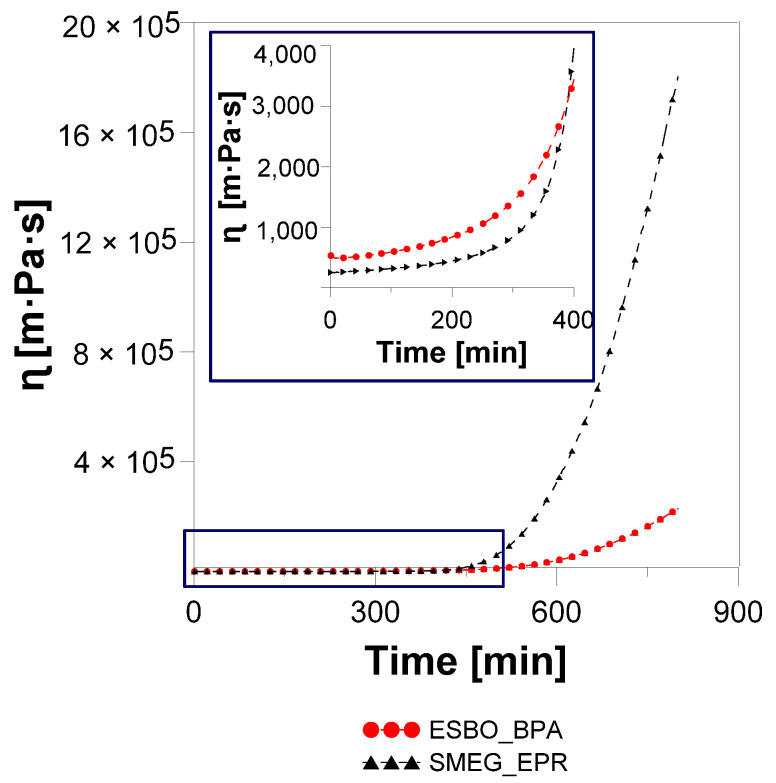
The course of changes in dynamic viscosity during the polyaddition reaction of modified soybean oil with BPA or EPR 0162 conducted in bulk, via the fusion process.

Compared with the ESBO_BPA synthesis, in the first stage (the so-called viscoelastic fluid stage), the SMEG_EPR system is characterized by lower values of G′ and G″, which indicates lower elasticity and lower viscosity of the hydroxylated soybean oil system subjected to the sinusoidal deformation. Compared to 1.7 × 10^−4^ Pa and 3.33 Pa for the ESBO_BPA, we recorded an increase in the value of G′ from 9.8 × 10^−5^ and G″ from 1.57 Pa. An observed phenomenon is related to the lower dynamic complex viscosity, which was recorded at a temperature of 160 °C at the beginning of the measurements of dynamic mechanical properties. For the process SMEG_EPR we recorded η_SMEG_EPR_* = 160.0 mPa s, while for the ESBO_BPA polyaddition product η_EOS_BPA_* = 250.3 mPa·s, respectively. Additionally, based on the analysis of the course of changes of rheological parameters for both polyaddition products (Figure 8 and Figure 9), it was found that point tg δ = 1 for the SMEG_EPR system was achieved much earlier than in the case of the ESBO_BPA (after t = 410 min for SMEG_EPR and t = 490 min for ESBO_BPA, respectively). At the same time, in the case of the SMEG_EPR synthesis, carried out in the flask in the laboratory, the determined content of epoxy groups overtime of 6.8 h (t = 410 min) was far away from the theoretical value, which was calculated for the gel point (*EV*_(*at* 6.8h)_ = 0.249 mol/100 g). In the case of reaction performed in the flask, the final polyaddition product in a form of a viscous liquid with epoxy groups content at the level of *EV* = 0.113 mol/100 g was obtained within 24 h. Additionally, for that process, after crossing the gel point, we recorded a larger increase in the value of G′ and finally higher values of the storage modulus. Thus, the SMEG_EPR final product shows much greater rigidity than the ESBO_BPA (for SMEG_EPR G′ = 1.1 × 10^4^ Pa compared to G′ = 1.8 × 10^3^ Pa for the system ESBO_BPA), as additionally evidenced by much higher values of dynamic viscosity for samples of the final polyaddition products (Figure 11).

Polyaddition products at t = 800 min of conducting the process are characterized by the dynamic viscosity of: 2.3 × 10^2^ and 1.8 × 10^3^ Pa·s, respectively for ESBO_BPA and SMEG_EPR.

## 4. Conclusions

The final products ESBO_BPA and SMEG_EPR, obtained during the polyaddition process of modified soybean oil and BPA or low-molecular-weight epoxy resin, which are characterized by a similar content of epoxy groups show significant differences in viscosity. Meaningfully higher viscosity was registered for the system based on hydroxylated soybean oil. Simultaneously, it was found that observed differences in viscosity significantly influenced the course of changes in characteristic dynamic mechanical properties of analyzed systems. It is worth emphasizing that only the correlation of the results of (*i*) the analysis of the content of functional groups, determining the potentially greatest degree of the conversion at the moment, which is the closest to the gelation point, as well as (*ii*) the expected gel time of the analyzed reaction system gives a full characterization of the obtained product. Moreover, it is the basis for the prediction of the degree of conversion of substrates at the point of the gel and performing the synthesis of the material, which after cross-linking using the properly selected hardener will be characterized by predetermined properties, such as, e.g., tensile, flexural, and compression strength, as well as hardness and toughness.
